# The Effect of Clonazepam on Restless Legs Syndrome in a Pregnant Woman: A Case Report of a Patient with Six Previous Pregnancies Accompanied by This Syndrome

**DOI:** 10.3390/jcm15124549

**Published:** 2026-06-11

**Authors:** Srđana Telarović, Lucija Čondić Jurjević

**Affiliations:** 1 School of Medicine, University of Zagreb, Salata 3b, 10000 Zagreb, Croatia; srdjana.telarovic@gmail.com; 2 Department of Neurology, University Hospital Centre Zagreb, Kišpatićeva 12, 10000 Zagreb, Croatia; 3 Vuk Vrhovac University Clinic, Clinical Hospital Merkur, Dugi Dol 4a, 10000 Zagreb, Croatia

**Keywords:** restless legs syndrome, multiparous pregnancy, clonazepam

## Abstract

**Background**: Restless legs syndrome (RLS) is very common among pregnant women; its prevalence corresponds to parity and occurs more frequently in the later stages of pregnancy. It is associated with numerous adverse pregnancy outcomes such as an increased risk of Caesarean section, preeclampsia, insomnia, and depression. Medical treatment of RLS during pregnancy is challenging considering the risks to both the mother and fetus. **Case Description**: In this report, we describe the case of a 44-year-old, seventh-time multiparous woman with a positive family history of RLS who presented with severe symptoms in her 24th week of pregnancy. She has had symptoms of RLS in every pregnancy thus far, usually starting in the second trimester, with an ineffective therapeutic response to diazepam and uncomfortable sensations, which usually resolve spontaneously a few weeks after delivery. After replacing diazepam with low-dose clonazepam, the patient reported complete disappearance of unpleasant sensations in her legs, with a significant improvement in her quality of life. **Conclusions**: A low dose of clonazepam is a good therapeutic option for the treatment of RLS during pregnancy and, if necessary, can also be considered the therapy of choice during lactation.

## 1. Background

Restless legs syndrome (RLS) is a movement disorder characterized by unpleasant sensations in the legs that lead to a strong urge to move the legs. The diagnosis was based on the criteria proposed by the International RLS Study Group [1]. The recent studies reveal a global prevalence of 21.9% for RLS among pregnant women [2]. The prevalence of RLS corresponds to parity and occurs more frequently in the second and third trimesters of pregnancy [3]. In most cases, symptoms disappear within a few weeks to one month postpartum [4]. RLS is a common cause of insomnia during pregnancy, and some studies have associated it with numerous adverse pregnancy outcomes, such as increased risk of Caesarean section, preeclampsia and depression [5]. In this article, we report the case of a multiparous pregnant woman with severe RLS who was treated with a low dose of clonazepam and had a good clinical outcome. The patient provided informed consent for participation, and the article was written in accordance with the principles of the Declaration of Helsinki with approval from competent ethical institutions.

## 2. Case Description

A 44-year-old woman in her 24th week of pregnancy was referred to a neurologist specializing in movement disorders by her obstetric team because of severe symptoms of RLS that occurred at the beginning of the second trimester. She described the symptoms as burning sensations in her legs that occurred in the evening and forced her to move constantly, which greatly disrupted her quality of sleep and life. A lower extremity Doppler ultrasound was performed, which successfully ruled out deep vein thrombosis. The diagnosis of RLS was then established via clinical examination using the international IRLSSG criteria, effectively excluding other pregnancy-related mimics, such as nocturnal leg cramps and peripheral edema. On the International Restless Legs Syndrome Study Group (IRLSSG) scale [6] she scored 25 points, classifying the symptom severity as severe. This is the patient’s seventh pregnancy in a row, and she states that she has had these symptoms in every pregnancy thus far, usually starting in the second trimester, with relief of symptoms a few weeks after delivery. All her previous pregnancies were without complications, and she delivered vaginally to all six children full term. The Apgar score of all six children was 10/10, with normal birth weights as follows: male, 3300 g; female, 2900 g; female, 3750 g; female, 3500 g; male, 3300 g; male, 4000 g. There is no record or evidence in the patient’s medical history of complications like preterm birth, birth trauma, neonatal intensive care unit (NICU) admission, hypoxic-ischemic encephalopathy, or infections. The patient’s mother and sister also suffered from restless legs syndrome, and she denied any other illnesses in the family. Her medical history was insignificant, she did not suffer from anemia or kidney disease, and there was no history of smoking, alcohol consumption, or recreational drug use before or during pregnancy. She did not receive any blood transfusions or oral or parenteral iron supplementation. Neurological and physical examination results were unremarkable. Her blood pressure and electrocardiographic findings were within normal limits. Laboratory findings, repeated several times during pregnancy, were normal; the baseline values are listed in Table 1.

Due to the borderline value of vitamin D, oral supplementation was administered. Non-pharmacological measures (such as avoidance of caffeine, massage, and moderate-intensity exercise) were minimally effective in alleviating her symptoms. According to the obstetrician’s recommendation, she was taking 10 mg diazepam at bedtime, which she occasionally used in previous pregnancies, but without any effect. Apart from prenatal vitamin intake, the patient did not take any other over-the-counter supplements. After consultation with a gynecologist and clinical pharmacologist, diazepam was substituted with a minimum dose of clonazepam (0.25 mg at bedtime for the first two days, and thereafter 0.5 mg), and she was scheduled for a follow-up examination within seven days. On follow-up examination, the patient reported a significant reduction in symptoms with good tolerability to the drug, and she did not report any side effects of the therapy. Her IRLSSG scale score improved significantly, shifting to the moderate symptom category. The patient was scheduled for a follow-up examination at four weeks. At the second follow-up examination, the patient reported further relief of symptoms, with significant improvement in sleep quality and no drug side effects, with her IRLSSG scale score indicating mild symptoms. She continued with regular obstetrician checkups, and a follow-up examination with a neurologist was scheduled for six–eight weeks. Regarding the pregnancy outcome, delivery occurred at 37 weeks of gestational age via vaginal delivery, resulting in a healthy newborn weighing 3310 g and measuring 49 cm with Apgar scores of 10 at 1 and 5 min; both the maternal and neonatal postpartum courses were uneventful.

## 3. Discussion

RLS is a common sleep disorder in pregnancy, and it has been shown to be associated with numerous adverse pregnancy outcomes. It is important to recognize this syndrome as early as possible and start treatment promptly, as well as to educate pregnant women about the importance of sleep hygiene and changing lifestyle habits (moderate physical activity, reduction of caffeinated drinks, smoking cessation, going to bed, and waking up at approximately the same time). Due to the frequent occurrence of anemia during pregnancy, iron replacement therapy should always be considered [7].

Medical treatment of RLS during pregnancy is challenging considering the risks to the mother and fetus; however, carbidopa-levodopa or low-dose clonazepam may be used in select patients [8,9,10,11]. Although some studies have associated clonazepam use during pregnancy with several potential fetal and neonatal risks, including miscarriage, fetal growth restriction, preterm birth, and ‘floppy infant syndrome’ [12], low-dose clonazepam is often regarded as a manageable therapeutic option during pregnancy. The therapeutic mechanism of clonazepam in RLS involves enhancing GABA-A receptor-mediated central inhibition, which reduces sensorimotor excitability. Additionally, as a long-acting benzodiazepine, it stabilizes sleep architecture by significantly decreasing sleep fragmentation and micro-arousals caused by periodic limb movements [13].

Most studies have demonstrated that the use of low-dose clonazepam during pregnancy does not appear to be directly related to any serious maternal or obstetric complications during pregnancy, labor, or delivery. Furthermore, there is no evidence of neonatal toxicity or withdrawal syndromes in infants born to mothers who took clonazepam during pregnancy [14,15,16]. These consistent observations across similar published cases reinforce the clinical utility of low-dose clonazepam when initial non-pharmacological approaches fail. While this case report inherently provides a lower level of clinical evidence than randomized trials, it offers crucial real-world data when clinical trials are ethically restricted in pregnant populations, bridging the gap between rigid guidelines and individual clinical practice for severe RLS.

## 4. Conclusions

In our patient, clonazepam proved to be a good choice for relieving the symptoms of RLS, and using a minimal dose of the drug reduced the possibility of side effects. In cases of persistent symptoms after delivery, low doses of clonazepam can also be considered a potential therapeutic option during lactation, after carefully weighing the risks and benefits [16]. The comprehensive and proper treatment of pregnant patients often requires the cooperation of multiple specialists.

## Figures and Tables

**Table 1:** Baseline laboratory values.

Test Result	Value	Reference Range
hemoglobin	135	119–157 g/L
hematocrit	0.396	0.356–0.470 L/L
red blood cell count	4.31	3.86–5.08 × 10^12^/L
mean corpuscular volume	91.9	83–97.2 fL
serum iron	120	50–170 mcg/dL
total iron-binding capacity	310	250–450 mcg/dL
ferritin	150	20–200 ng/mL
AST	20	10–30 U/L
creatinine	0.86	0.5–1.1 mg/dL
glucose	85	70–110 mg/dL
sodium	139	136–145 mEq/L
potassium	3.8	3.5–5.0 mEq/L
total serum calcium	9.1	8.2–10.2 mg/dL
magnesium	1.8	1.3–2.1 mEq/L
TSH	2.8	0.5–5.0 mIU/L
vitamin D	20.83	deficient: below 20 ng/mL

## Data Availability

Data are contained within the article.

## References

[1] Allen R.P., Picchietti D.L., Garcia-Borreguero D., Ondo W.G., Walters A.S., Winkelman J.W., Zucconi M., Ferri R., Trenkwalder C., Lee H.B. (2014). Restless legs syndrome/Willis-Ekbom disease diagnostic criteria: Updated International Restless Legs Syndrome Study Group (IRLSSG) consensus criteria–history, rationale, description, and significance. Sleep Med..

[2] Altalbawy F.M.A., Kareem M., Sanghvi G., R R., Kashyap A., Indumathi S.M., Panigrahi R., Kubaev A., Joshi K.K. (2026). Prevalence of restless legs syndrome during pregnancy: A systematic review and meta-analysis. Sleep Med..

[3] Gupta R., Dhyani M., Kendzerska T., Pandi-Perumal S.R., BaHammam A.S., Srivanitchapoom P., Pandey S., Hallett M. (2016). Restless legs syndrome and pregnancy: Prevalence, possible pathophysiological mechanisms and treatment. Acta Neurol. Scand..

[4] Picchietti D.L., Hensley J.G., Bainbridge J.L., A Lee K., Manconi M., A McGregor J., Silver R.M., Trenkwalder C., Walters A.S. (2015). International Restless Legs Syndrome Study Group (IRLSSG). Consensus clinical practice guidelines for the diagnosis and treatment of restless legs syndrome/Willis-Ekbom disease during pregnancy and lactation. Sleep Med. Rev..

[5] Dunietz G.L., Lisabeth L.D., Shedden K., Shamim-Uzzaman Q.A., Bullough A.S., Chames M.C., Bowden M.F., O’BRien L.M. (2017). Restless Legs Syndrome and Sleep-Wake Disturbances in Pregnancy. J. Clin. Sleep Med..

[6] The International Restless Legs Syndrome Study Group (2003). Validation of the International restless legs syndrome study group rating scale for restless legs syndrome. Sleep Med..

[7] Schneider J., Krafft A., Manconi M., Hübner A., Baumann C., Werth E., Gyr T., Bassetti C. (2015). Open-label study of the efficacy and safety of intravenous ferric carboxymaltose in pregnant women with restless legs syndrome. Sleep Med..

[8] Jahani Kondori M., Kolla B.P., Moore K.M., Mansukhani M.P. (2020). Management of Restless Legs Syndrome in Pregnancy and Lactation. J. Prim. Care Community Health.

[9] Saletu M., Anderer P., Saletu-Zyhlarz G., Prause W., Semler B., Zoghlami A., Gruber G., Hauer C., Saletu B. (2001). Restless legs syndrome (RLS) and periodic limb movement disorder (PLMD): Acute placebo-controlled sleep laboratory studies with clonazepam. Eur. Neuropsychopharmacol..

[10] Lin A.E., Peller A.J., Westgate M.N., Houde K., Franz A., Holmes L.B. (2004). Clonazepam use in pregnancy and the risk of malformations. Birth Defects Res. A Clin. Mol. Teratol..

[11] Srivanitchapoom P., Pandey S., Hallett M. (2014). Restless legs syndrome and pregnancy: A review. Park. Relat. Disord..

[12] Grigoriadis S., Graves L., Peer M., Mamisashvili L., Ruthirakuhan M., Chan P., Hennawy M., Parikh S., Vigod S.N., Dennis C.-L. (2020). Pregnancy and Delivery Outcomes Following Benzodiazepine Exposure: A Systematic Review and Meta-analysis. Can. J. Psychiatry.

[13] Garcia-Borreguero D., Silber M.H., Winkelman J.W., Högl B., Bainbridge J., Buchfuhrer M., Hadjigeorgiou G., Inoue Y., Manconi M., Oertel W. (2016). Guidelines for the first-line treatment of restless legs syndrome/Willis-Ekbom disease, prevention and treatment of dopaminergic augmentation: A combined task force of the IRLSSG, EURLSSG, and the RLS-foundation. Sleep Med..

[14] Weinstock L., Cohen L.S., Bailey J.W., Blatman R., Rosenbaum J.F. (2001). Obstetrical and neonatal outcome following clonazepam use during pregnancy: A case series. Psychother. Psychosom..

[15] Munger Clary H.M. (2024). Caution: Benzodiazepines in Pregnancy and Risk of Adverse Perinatal Outcomes. Epilepsy Curr..

[16] Kelly L.E., Poon S., Madadi P., Koren G. (2012). Neonatal benzodiazepines exposure during breastfeeding. J. Pediatr..

